# Naturally Acquired Antibody Response to Malaria Transmission Blocking Vaccine Candidate Pvs230 Domain 1

**DOI:** 10.3389/fimmu.2019.02295

**Published:** 2019-10-04

**Authors:** Bergeline C. Nguemwo Tentokam, Chanaki Amaratunga, Nada A. H. Alani, Nicholas J. MacDonald, David L. Narum, Nichole D. Salinas, Jennifer L. Kwan, Seila Suon, Sokunthea Sreng, Dhelio Batista Pereira, Niraj H. Tolia, Ricardo T. Fujiwara, Lilian L. Bueno, Patrick E. Duffy, Camila H. Coelho

**Affiliations:** ^1^Laboratory of Malaria Immunology and Vaccinology, National Institute of Allergy and Infectious Diseases, National Institutes of Health (NIH), Bethesda, MD, United States; ^2^Laboratory of Malaria and Vector Research, National Institute of Allergy and Infectious Diseases, National Institutes of Health (NIH), Rockville, MD, United States; ^3^Laboratory of Clinical Immunology and Microbiology, National Institute of Allergy and Infectious Diseases, National Institutes of Health (NIH), Bethesda, MD, United States; ^4^National Center for Parasitology, Entomology and Malaria Control, Phnom Penh, Cambodia; ^5^Centro de Pesquisa em Medicina Tropical, Porto Velho, Brazil; ^6^Department of Parasitology, Federal University of Minas Gerais, Belo Horizonte, Brazil

**Keywords:** malaria, *Plasmodium vivax*, Pvs230, transmission-blocking vaccine, seroreactivity

## Abstract

*Plasmodium vivax* malaria incidence has increased in Latin America and Asia and is responsible for nearly 74.1% of malaria cases in Latin America. Immune responses to *P. vivax* are less well characterized than those to *P. falciparum*, partly because *P. vivax* is more difficult to cultivate in the laboratory. While antibodies are known to play an important role in *P. vivax* disease control, few studies have evaluated responses to *P. vivax* sexual stage antigens. We collected sera or plasma samples from *P. vivax*-infected subjects from Brazil (*n* = 70) and Cambodia (*n* = 79) to assess antibody responses to domain 1 of the gametocyte/gamete stage protein Pvs230 (Pvs230D1M). We found that 27.1% (19/70) and 26.6% (21/79) of subjects from Brazil and Cambodia, respectively, presented with detectable antibody responses to Pvs230D1M antigen. The most frequent subclasses elicited in response to Pvs230D1M were IgG1 and IgG3. Although age did not correlate significantly with Pvs230D1M antibody levels overall, we observed significant differences between age strata. Hemoglobin concentration inversely correlated with Pvs230D1M antibody levels in Brazil, but not in Cambodia. Additionally, we analyzed the antibody response against Pfs230D1M, the *P. falciparum* ortholog of Pvs230D1M. We detected antibodies to Pfs230D1M in 7.2 and 16.5% of Brazilian and Cambodian *P. vivax*-infected subjects. Depletion of Pvs230D1M IgG did not impair the response to Pfs230D1M, suggesting pre-exposure to *P. falciparum*, or co-infection. We also analyzed IgG responses to sporozoite protein PvCSP (11.4 and 41.8% in Brazil and Cambodia, respectively) and to merozoite protein PvDBP-RII (67.1 and 48.1% in Brazil and Cambodia, respectively), whose titers also inversely correlated with hemoglobin concentration only in Brazil. These data establish patterns of seroreactivity to sexual stage Pvs230D1M and show similar antibody responses among *P. vivax*-infected subjects from regions of differing transmission intensity in Brazil and Cambodia.

## Introduction

Malaria is a vector-borne infectious disease caused by the *Plasmodium* protozoan parasite. Over 200 million people suffer malaria episodes every year, primarily in tropical low-income settings, and pregnant women and children are particularly vulnerable to severe disease (1). Malaria eradication is a global priority, and an efficacious vaccine could strengthen current control efforts and enable elimination strategies. Vaccine development depends on the understanding of protective immunity, and it is fundamental to characterize immune responses to infection in a natural setting. While much research has focused on *P. falciparum*, the species causing most morbidity and mortality, immune responses to *P. vivax* infection are less well studied.

In 2017, Brazil reported an increase in malaria incidence rate that contributed to 25% of malaria cases in all of Latin America, the majority of which (74.1%) were caused by *P. vivax* infection (1). But not only the Americas are affected by vivax malaria. Cambodia, in Asia, is particularly affected by malaria, reporting a 98% increase in clinical cases between 2016 and 2017 (1). Neither Cambodia nor Brazil are expected to meet the goal of 40% malaria reduction by 2020, thus, both countries require additional strategies to control and prevent malaria infection and transmission. Importantly, vivax malaria is a global issue (2) and an increase in the number of *P. vivax* cases has been recently reported in Africa (3–6).

Prevention tools that target the sexual stages of parasites may be critical to reduce disease incidence in locations where transmission rates are increasing. Transmission to the next vulnerable human can be halted by disrupting the development of the sexual stage parasite in the mosquito, the basis for the development of transmission-blocking vaccines (TBV) (7). Naturally acquired immunity to *P. falciparum* TBV candidates is well characterized (8–10) and TBVs for *P. falciparum* are currently in pre-clinical and clinical trials (11–14). However, *P. vivax* TBV candidates are less advanced. To date, only Pvs25, a post-fertilization antigen present on the surface of *Plasmodium* zygotes and ookinetes, has been evaluated as a human vaccine targeting *P. vivax* sexual stages (15, 16). Although Pvs25 immunization has shown promising results in mice, achieving durable anti-Pvs25 antibody responses remains challenging and no boosting effect of natural exposure is expected, thus multiple vaccinations may be required. We hypothesize that the development of a vaccine able to target a pre-fertilization antigen may benefit from boosting during natural infections and thereby reduce transmission more effectively. Pvs230 (the ortholog of the *P. falciparum* Pfs230) is a pre-fertilization gametocyte/gamete antigen in *P. vivax* parasites with a low level of polymorphism worldwide (17), making it a promising target for TBV strategies in Asia and Latin America. Studies have explored Pvs230 TBV candidacy by assessing mouse antisera raised against four domains of the Pvs230 protein (18), but prevalence of anti-Pvs230 antibodies during naturally acquired infection in humans has never been assessed.

Here, we evaluated seroprevalence to the first domain of the sexual stage antigen Pvs230 (Pvs230D1M) in *P. vivax*-infected subjects in malaria-endemic areas of Brazil and Cambodia. Our results can inform future strategies to develop Pvs230D1M as a transmission-blocking vaccine.

## Methods

### Ethical Approvals, Study Sites, Patients, and Sample Collections

Prior approval of the clinical study protocols was obtained from the Centro de Pesquisa em Medicina Tropical (CAAEs: 0008.0.046.000-11, 0449.0.203.000-09) and the Ethics Committee of the Federal University of Minas Gerais (CAAE: 27466214.0.0000.5149) in Brazil, and the National Ethics Committee for Human Research (ClinicalTrials.gov Identifier: NCT00663546) in Cambodia, and by the Institutional Review Board, NIAID, NIH. Written informed consent was obtained from each participant.

**Figure 1:**
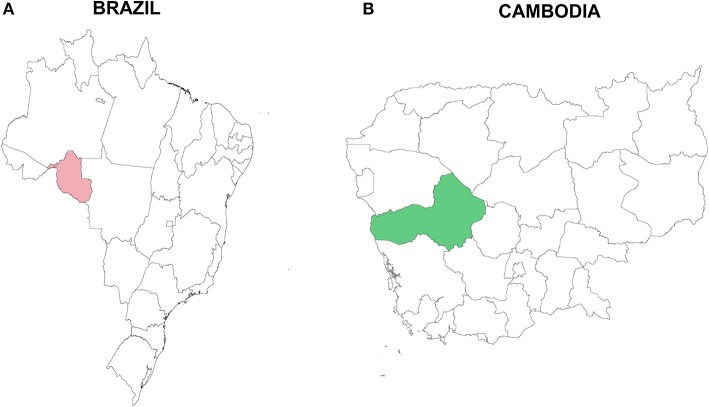
Study sites in **(A)** Rondônia state, Brazil, South America (pink), and **(B)** Pursat province, Cambodia, Asia (green). Samples were collected in Brazil in 2011 and 2014 and in Cambodia during 2010.

Seventy serum samples were obtained from adults from Rondônia state, Brazil during 2011 and 2014, and 79 plasma samples were obtained from children and adults from Pursat province, Cambodia in 2010 (Table 1, Figure 1) when they presented with acute *P. vivax* infection. Presence of *P. vivax* parasites was diagnosed by microscopy and absence of *P. falciparum* parasites was also established; gametocytes were not separately documented by microscopy and are hence not available for analyses. Sera (Brazil) or plasma (Cambodia) were frozen and transported to NIH in Rockville, USA, for further analysis. Additional information on patients from this study is presented in Table S1.

**Table 1:** Main demographic characteristics of study participants in Brazil and Cambodia.

		**Brazil**	**Cambodia**
Sex^*^	Male (%)	53 (76.8%)	61 (77.2%)
	Female (%)	16 (23.2%)	18 (22.8%)
Age (years)^**^	1–9 years	0 (0%)	8 (10.1%)
	10–19 years	0 (0%)	26 (32.9%)
	>20 years	67 (100.0%)	45 (57.0%)

*Samples were predominantly from male subjects. In Brazil, only samples from adults were collected. Additional demographic information is provided in Table S1*.

* *Information not available for 1 Brazilian subject*.

** *Information not available for 3 Brazilian subjects*.

### Pvs230D1M, Pfs230D1M, PvDBP-RII, and PvCSP ELISA

Antibody responses against Pfs230D1M, Pvs230D1M, PvDBP-RII (*P. vivax* Duffy Binding Protein Region II) and PvCSP *(P. vivax* Circumsporozoite Protein) recombinant antigens were determined by enzyme-linked immunosorbent assay (ELISA). Pfs230D1M was expressed in *Pichia pastoris* as previously described (19). Details for the production and purity of Pvs230D1M (Sal-1, NCBI reference sequence XP_001613020.1) and PvCSP (CSP31VK210, NCBI reference KT588189.1), which were also produced in *P. pastoris*, will be reported elsewhere [manuscript in preparation]. PvDBP-RII was expressed in *E. coli* BL-21 cells and refolded as previously described (20–23).

**Figure 2:**
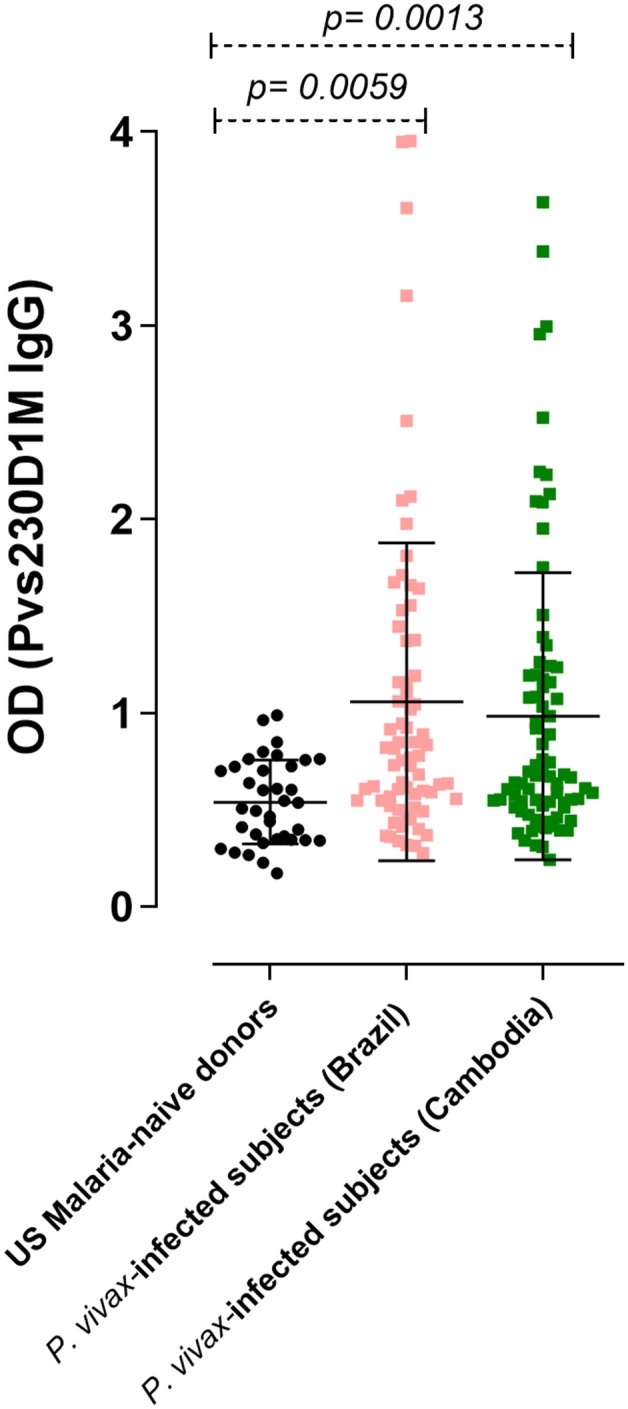
IgG response against Pvs230D1M in *P. vivax*-infected subjects, determined by ELISA. The seroprevalence of antibodies with specificity for Pvs230D1M in Brazil was 27.1% (19/70 subjects), and in Cambodia 26.6% (21/79 subjects). Cut-off value (1.18) was calculated based on mean control + 3 standard deviations. One-Way ANOVA followed by multiple comparisons was used for this analysis and results are displayed as mean ± SD.

**Figure 3:**
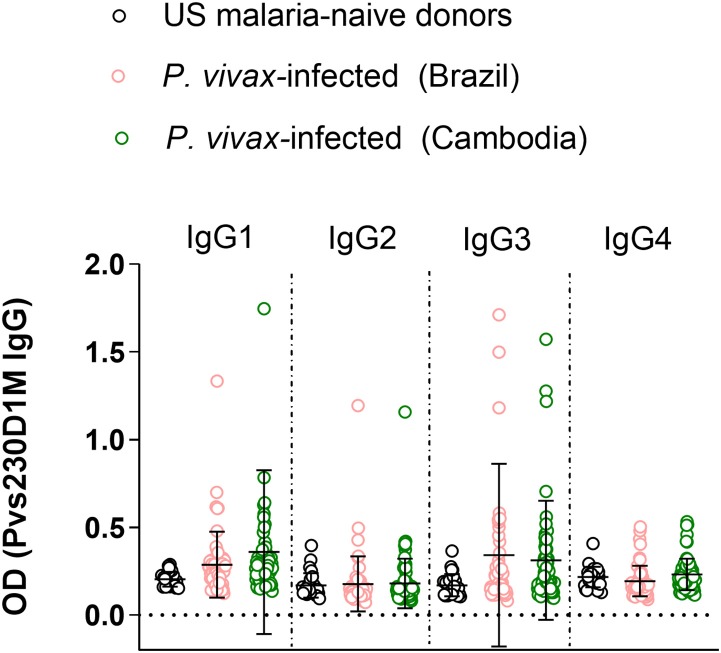
Immunoglobulin G subclass responses to Pvs230D1M in Brazil and Cambodia. Healthy malaria-naïve donor sera were used to define the background for each subclass, and cut-off was calculated based on mean control + 3 standard deviations. IgG3 and IgG1 responses were predominant with limited IgG2 and no IgG4 response. One-Way ANOVA followed by multiple comparisons was used for this analysis and results are displayed as mean ± SD.

Immulon® 4HBX plates were coated with 1 μg/mL of recombinant antigens, then incubated overnight at 4°C. Coated plates were blocked with 320 μL of buffer containing 5% skim milk in Tris-buffered saline (TBS) for 2 h at room temperature (RT), and washed four times with 1X Tween-TBS. After establishing minimum serum dilutions to detect reactivity against individual antigens in pilot studies, plasma, or serum samples (diluted 1:10 for Pvs230D1M, 1:100 for Pfs230D1M, 1:50 for PvDBP-RII, and 1:250 for PvCSP in blocking buffer) were added to antigen-coated wells in duplicate and incubated for 2 h at RT. Plates were washed and incubated with 100 μL anti-human IgG (1:2000 dilution; SeraCare: KPL) for 2 h at RT. The plates were washed and subsequently incubated in the dark for 30 min at RT with a colorimetric substrate (p-nitrophenyl phosphate; Sigma). Absorbances (405 and 650 nm) were measured using SoftMax Pro7 ELISA reader (Molecular Devices). The cut-off to define positivity was based on the average optical density (OD) from 36 non-immune serum samples from USA donors (negative controls), whose values did not differ significantly between experiments (p>0.9) and hence OD values for controls from different assays were combined for (Figures 2, 3, **7**). The cut-off for positivity was calculated as the mean OD of negative controls plus 3 standard deviations.

### Detection of Pvs230D1M IgG Subclasses by ELISA

Immulon® 4HBX plates were coated with 5 μg/mL of recombinant Pvs230D1M antigens, then incubated overnight at 4°C. Coated plates were blocked with 320 μL of buffer containing 5% skim milk in Tris-buffered saline (TBS) for 2 h at RT, and washed four times with 1X Tween-TBS. Plasma or serum samples were diluted 1:10 in blocking buffer and were added to antigen-coated wells in duplicate and incubated for 2 h at RT. IgG subclasses were detected using the following antibodies: mouse anti-human IgG1 Fc-AP, mouse anti-human IgG2 Fc-AP, mouse anti-human IgG3 Hinge-AP, and mouse anti-human IgG4 Fc-AP from Southern Biotech for 2 h at RT. All these antibodies were diluted 1:750 in blocking buffer. The plates were washed and subsequently incubated in the dark for 30 min at RT with a colorimetric substrate (p-nitrophenyl phosphate; Sigma). Absorbances (405 nm and 650 nm) were measured using SoftMax Pro7 ELISA reader (Molecular Devices). The cut-off to define positivity was based on the average optical density (OD) from 36 non-immune sera samples from USA donors (negative controls). The cut-off for positivity was calculated as the mean OD of negative controls plus 3 standard deviations. A sample was considered positive if background-adjusted OD was above the cut-off value.

### Antibody Depletion and ELISA

Immulon® 4HBX plates were coated with 1 μg/ml of recombinant Pfs230D1M, Pvs230D1M or PvDBP-RII respectively, incubated overnight at 4°C, blocked for 2 h at RT, and washed. Thereafter, 100 μl of sample (diluted 1:50) were added to Pvs230D1M-coated wells and incubated for 1 h at RT. The unbound material from the Pvs230D1M coated plate was collected and transferred into another well coated with same antigen. After the third transfer, depleted antibodies were transferred to Pfs230D1M- or PvDBP-RII-coated plates and incubated for 1 h at RT, and further processing was performed as described above. IgG responses were considered cross-reactive if preincubation with Pvs230D1M resulted in reduced antibody reactivity in Pfs230D1M ELISA. If preincubation with Pvs230D1M did not reduce antibody reactivity in the Pfs230D1M ELISA, the IgG response against Pfs230D1M in *P. vivax*-infected subjects was presumed to be due to *vivax*/*falciparum* co-infection or to pre-exposure to *P. falciparum*. Pvs230D1M-depleted sera were transferred to PvDBP-RII-coated plates, to confirm that the depletion or reduction found in Pvs230D1M ELISA was specific for antibodies targeting Pvs230D1M (Figure S3). For this experiment sera from 26 USA donors were used as negative controls.

### Statistical Analyses

**Figure 4:**
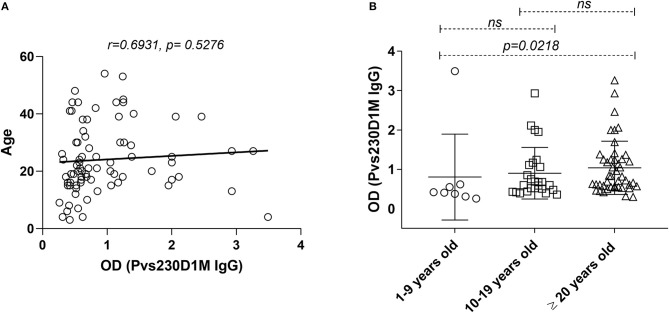
Pvs230D1M IgG responses do not correlate with age in Cambodia but increase significantly within age strata. **(A)** Pearson correlation and Linear regression, comparing Pvs230D1M antibody titers and age. **(B)** Seroprevalence for Pvs230D1M IgG (mean ± SD) was increased with age strata in Cambodian subjects: Pvs230D1M IgG response detected in 1.3% of 1–9 year old children (*N* = 8), 6.3% in 10–19 year old children (*N* = 26), and 19% in adults 20 years and older (*N* = 45). Kruskal-Wallis statistic test was used for the comparisons.

**Figure 5:**
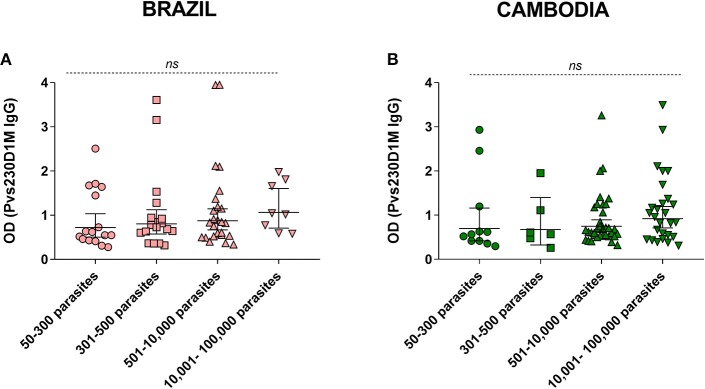
Parasitemia levels in comparison to Pvs230D1M IgG titers in **(A)** Brazil and **(B)** Cambodia. Kruskal-Wallis analysis revealed no significant difference between the groups. Detailed information on parasitemia levels for each subject is reported in Table S1.

Statistical analyses were performed using GraphPad Prism software (GraphPad8). Analyses were performed using data from two independent experiments. We considered serum reactivity levels above a maximum threshold of 3 standard deviations from the geometric mean for the study population to be unreliable, and hence excluded one Brazilian subject from the analyses. Correlation analyses were tested using logistic-regression analysis. One-Way ANOVA followed by multiple comparisons test was employed to compare different groups, when applicable. For Figures 4, 5, Kruskal-Wallis test was performed. Significance level used was *p* < 0.05 for all statistical analyses.

### Map Design

Maps were created using *maptools* and *raster* packages and plotted using *ggplot2* package of the R software (http://www.r-project.org, version 3.5.3).

## Results

### Anti-Pvs230D1M Antibody Response Is Induced During Malaria Infection

To assess humoral response to sexual stage antigens in subjects living in areas of malaria transmission, sera/plasma samples obtained from patients presenting with acute *P. vivax* infection diagnosed by blood smear microscopy were examined for IgG levels against Pvs230D1M. Sera from 36 healthy non-immune USA donors were used as negative controls to determine the cut-off OD value (1.18). The seroprevalence of IgG antibodies with specificity for Pvs230D1M was 27.1% in Brazil (19/70 samples) and 26.6% in Cambodia (21/79 samples) (Figure 2).

### IgG1 and IgG3 Are the Most Prevalent IgG Subtype Responses to Pvs230D1M

To evaluate differential representation in immune response to Pvs230 among the four human IgG subtypes, we evaluated IgG1, 2, 3, and 4 responses against Pvs230D1M. Detectable IgG3 levels (19.3% in Brazil and 20.6% in Cambodia) and IgG1 levels (10.5% in Brazil and 15.1% in Cambodia) were most frequent, with limited IgG2 responses (5.3% in Brazil and 1.4% in Cambodia). The frequency of IgG4 responses was 0% at both study sites (Figure 3).

### Pvs230D1M IgG Response Increases With Age in Cambodian Subjects

Although no direct correlation was observed between age and Pvs230D1M IgG titers (Figure 4), a cumulative effect of age in Pvs230D1M antibody response was observed in Cambodia. Seroprevalence for Pvs230D1M was higher with increasing age strata in Cambodian subjects: 1.3% Pvs230D1M IgG responders among 1–9 year-olds; 6.3% among 10–19 year-olds; and 19.0% among 20 years old and above (1–9 years group vs. 20 years and older, *p* = 0.021, Figure 4). Pvs230D1M titers were not evaluated for correlation with age in Brazil since the median age was 45 years old and no samples were obtained from children.

### Anti-Pvs230D1M IgG Titers Do Not Correlate With Parasitemia Levels

We assessed the correlation of anti-Pvs230D1M specific antibodies with parasitemia levels. There was no significant association between Pvs230D1M IgG response and asexual parasitemia in subjects from Brazil (*p* = 0.38) or Cambodia (*p* = 0.43) (Figure 5).

### Increased Pvs230D1M IgG Titers Correlate With Decreased Hemoglobin in Brazil, but Not in Cambodia

**Figure 6:**
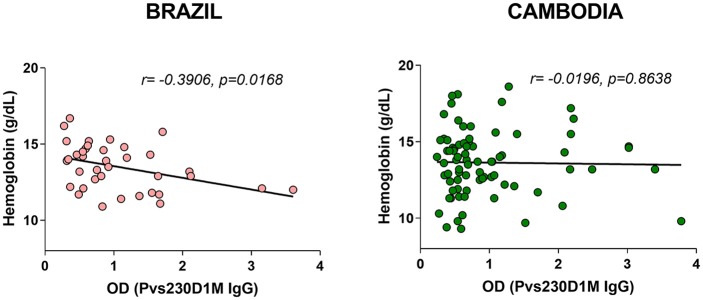
Pvs230D1M-specific antibody responses inversely correlate with hemoglobin levels in Brazil but not in Cambodia.

Despite its historical designation as “benign tertian malaria,” *P. vivax* has received increased attention as a cause of severe sequelae, including severe anemia (24, 25). We assessed whether *P. vivax* antibody levels are inversely correlated with hemoglobin levels, to support the hypothesis that anemia in either of these populations may be due to *P. vivax* infections. Antibodies elicited in response to Pvs230D1M were negatively correlated with hemoglobin levels in Brazilian subjects (*r* = −0.3906, *p* = 0.0168), but not in Cambodian subjects (Figure 6).

### Increased PvDBP-RII and PvCSP IgG Titers Correlate With Decreased Hemoglobin in Brazil

**Figure 7:**
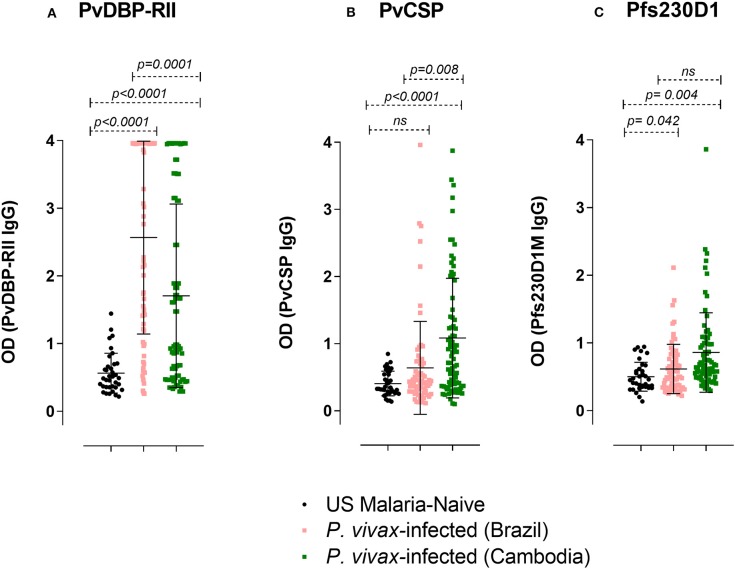
IgG ELISA levels against PvDBP-RII, PvCSP, and Pfs230D1M in *P. vivax-*infected subjects. The seroprevalence of IgG antibodies with specificity for **(A)** PvDBP-RII in Brazil was 67.1% (47/70 subjects) and in Cambodia 48.1% (38/79 subjects); **(B)** PvCSP in Brazil was 11.4% (8/70 subjects) and in Cambodia 41.8% (33/79 subjects); **(C)** Pfs230D1M in Brazil was 7.2% (5/69 subjects; the volume of one sample was insufficient for assay) and in Cambodia 16.5% (13/79). The cut-off levels for detection (1.44, 0.97, and 1.16 respectively for PvDBP-RII, PvCSP, and Pfs230D1M ELISA) were based on control mean + 3SD calculation. One-Way ANOVA followed by multiple comparisons was used for this analysis and results are displayed as mean ± SD.

**Figure 8:**
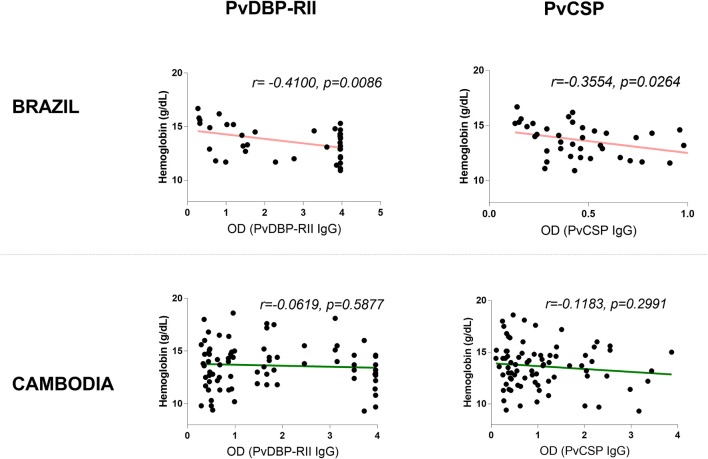
Correlation between antibodies elicited in response to blood-stage antigen (PvDBP-RII) or sporozoite-stage antigen (PvCSP) and hemoglobin levels in Brazil and Cambodia.

To assess whether the correlation of hemoglobin and antibody titers is specific to Pvs230D1M IgG, we analyzed the relationships with *P. vivax* proteins PvCSP (sporozoite stage protein) and PvDBP-RII (merozoite stage protein). The seroprevalence of antibodies with specificity for PvDBP-RII was 67.1% (47/70 samples) in Brazil and 48.1% (38/79 samples) in Cambodia, and for PvCSP 11.4% (8/70 samples) and 41.8% (33/79 samples) respectively (Figure 7). Hemoglobin levels negatively correlated with PvDBP-RII (*r* = −0.4100, *p* = 0.0086) and PvCSP (*r* = −0.3554, *p* = 0.0247) IgG titers in Brazil (Figure 8), but no correlations were seen in Cambodia, indicating that the relationships to hemoglobin are similar for seroreactivities against liver stage, blood stage, and sexual stage antigens within the two study populations. Antibody levels against liver and blood stage proteins did not significantly correlate with age in Brazil or Cambodia (Figure S1).

### Antibodies to Pfs230D1M, *P. falciparum* Ortholog of Pvs230D1M, in *P. vivax*-Infected Subjects

We investigated whether antibody responses during *P. vivax* infection might also be reactive against Pfs230D1M. We found that 7.2% of the sera from *P. vivax*-infected subjects from Brazil and 16.5% from Cambodia had detectable antibody against Pfs230D1M (Figure 7).

### Concurrent Antibody Responses to Pvs230D1M and Pfs230D1M Are Not Due to Shared Epitopes

**Figure 9:**
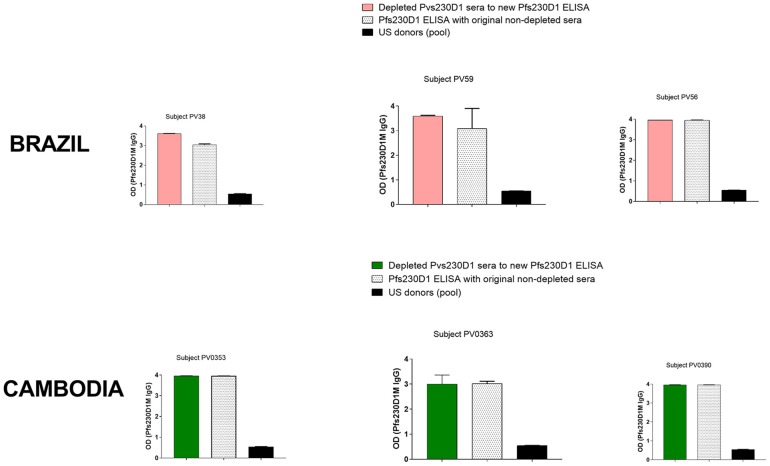
ELISA on Pvs230D1M-depleted sera. Pvs230D1M IgG levels were gradually reduced in sera by depletion assay (Figure S2), and then samples were submitted to ELISA against Pfs230D1M. Specificity of depletion was confirmed measuring antibody titers to PvDBP-RII in sera depleted for Pvs230 IgG (Figure S3).

Due to similarities between Pvs230D1M and Pfs230D1M protein sequences, and the fact that *P. falciparum* malaria cases are also present in Brazil and Cambodia, we examined whether subjects may have developed antibody responses to both Pvs230D1 and Pfs230D1. Three (4.3%) subjects in Brazil and six (7.6%) subjects in Cambodia presented with concurrent antibody responses to Pvs230D1 and Pfs230D1. We assayed Pvs230D1M-depleted sera to investigate whether Pfs230D1M titers resulted from cross-reactive epitopes with Pvs230D1M. After depletion assay to remove antibodies specifically generated against Pvs230 (Figure S2), Brazilian and Cambodian samples maintained IgG levels to Pfs230D1M comparable to pre-depletion levels (Figure 9), suggesting that responses were not due to cross-reactive epitopes. Pfs230D1M antibody titers may therefore be due to microscopically undetected co-infection with *P. falciparum* or previous exposure to *P. falciparum* in these populations.

## Discussion

Antibodies to sexual stage parasites can be induced in response to infection (9, 10, 26). Compared to *P. vivax*, the antibody response to *P. falciparum* sexual stages is better characterized and it is known that children and adults in endemic areas acquire an immune response to Pfs230 (8). However, the naturally acquired response to Pvs230 has not been characterized, despite *P. vivax* being responsible for the majority of malaria cases in Latin America and in Southeast Asia (1). Understanding adaptive immune responses to *P. vivax* antigens present in sexual-stage parasites in the mosquito and human host can contribute to development of transmission-blocking strategies. In the current study, the prevalence of antibodies against domain 1 of Pvs230 (Pvs230D1M) during *P. vivax* infection was 27.1% in Brazil and 26.6% in Cambodia. Similarly, previous studies have shown that the ortholog Pfs230 reacted to sera from 28.6% of malaria-exposed adults in an area of seasonal transmission in Burkina Faso and from 20.7% of *P. falciparum*-infected donors in a low endemic area of Tanzania (8, 10).

Although anti-Pfs230 antibody activity can be enhanced by complement (27, 28), information on IgG subclasses generated against Pvs230 in naturally infected humans has not been described. Previous work showed that sera from mice immunized with Pvs230 reduces the number of oocysts in midguts of mosquitoes fed with blood from *P. vivax*-infected subjects, and this reduction occurs in the presence or absence of complement (18). We evaluated whether the natural antibody response to Pvs230D1M in humans would be characterized by higher levels of complement-fixing IgG subclasses. In our analyses of Pv230D1M IgG subclass frequency, IgG1 and IgG3 were shown to be the predominant subclasses during malaria infection and these isotypes are known to fix complement (29–33). This suggests that the functional activity of naturally acquired anti-Pvs230 antibody might be enhanced by complement, but this requires further investigation.

Although the correlation between age and Pvs230D1M IgG was not statistically significant, Pvs230D1M-specific antibody titers in Cambodia differed (*p* = 0.021) between 1–9 year old subjects vs. subjects ≥20 years old. These results need to be interpreted in the context of characteristics of the study sites. In Pursat province, Cambodia, exposure to malaria frequently occurs as a result of occupation and exposure is low in children. A longitudinal study must be conducted with a larger number of samples collected from high and low endemic areas to confirm the age-cumulative effect of Pvs230 IgG response and its relationship with high or low transmission areas. Previous studies have suggested that IgG responses against sexual stage *P. falciparum* proteins do not increase with age (8–10, 26). For example, a study performed in a low *P. falciparum* transmission area in Tanzania did not reveal correlations between antibodies generated in response to Pfs230 or to Pfs48/45 and age (10).

*P. vivax* is associated with lower hemoglobin concentration and can cause severe malaria (34–37). Here, we found an inverse correlation between anti-Pvs230D1M antibody titers and hemoglobin levels in Brazil. Confirming that low levels of hemoglobin were due to malaria, we observed the same correlation with PvDBP-RII and PvCSP IgGs (elicited in response to blood stage and pre-erythrocytic stage parasites, respectively). PvDBP-RII titers were higher in Brazil than in Cambodia, supporting our hypothesis that exposure in Rondônia state may be higher than in Pursat province, Cambodia. Intriguingly, PvCSP titers were higher in Cambodia than in Brazil, which may be attributable to the fact that the PvCSP recombinant protein was based on a parasite strain isolated in Iran (VK210), a country in Asia, closer to Cambodia than to Brazil.

In Cambodia, no correlation was observed between antibody titers and hemoglobin levels. We hypothesize that high endemicity with more frequent infections in a region such as Brazil lowers hemoglobin levels and therefore negatively correlates with increased antibody levels, while low endemicity in a region such as Cambodia entails more sporadic infections with potentially less impact on hemoglobin levels. Of note, the ranges of hemoglobin levels were similar at the two study sites, as were the proportions of male and female subjects. Hemoglobin levels assessed prior to infection were not determined, since those samples were not available for this study.

We found no relationship of anti-Pvs230D1M antibody to level of parasitemia in Brazil (*r* = 0.060, *p* = 0.6469) or Cambodia (*r* = 0.1193, *p* = 0.2948). Data on gametocytemia were not collected at the time of blood smear microscopy and therefore are not available for analysis. In future, it will be of interest to perform a longitudinal study, to evaluate serologic parameters identified before, during and after infection and to correlate *P. vivax* sexual stage antibody responses to gametocyte carriage.

*P. vivax*-infected subjects from Brazil and Cambodia displayed antibodies against Pfs230D1M, the ortholog of Pvs230D1M in *P. falciparum*. ELISA on Pvs230D1M-depleted sera suggests that Pfs230D1M titers were produced in response to *P. falciparum* pre-exposure or co-infection. The Pfs230D1M IgG response was more frequent in Cambodia than Brazil, perhaps reflecting the greater proportion of malaria infections caused by *P. falciparum* in Cambodia vs. Brazil (58 vs. <10%) (1). Since *P. falciparum* infection is known to cause anemia (38, 39), a mixed infection could influence the correlation of hemoglobin with antibody titers. A limitation in our study was that the low volume of plasma samples precluded determination of functional activity of Pfs230-purified IgG in Standard Membrane Feeding Assay (SMFA) that assesses the reduction of *P. falciparum* parasite transmission to mosquitoes.

Our findings provide a first characterization of naturally acquired antibody responses to Pvs230 among *P. vivax*-infected subjects from regions of differing transmission intensity in Brazil and Cambodia.

## Data Availability Statement

Datasets generated for this study are included in the manuscript/Supplementary Files. Additional datasets are also available upon request.

## Ethics Statement

The studies involving human participants in Brazil were reviewed and approved by the Centro de Pesquisa em Medicina Tropical (CAAEs: 0008.0.046.000-11, 0449.0.203.000-09) and the Ethics Committee of the Federal University of Minas Gerais (CAAE: 27466214.0.0000.5149), Brazil. The human study in Cambodia was approved by the Institutional Review Board (IRB), NIAID, NIH, and National Ethics Committee for Human Research (NECHR), Cambodia (ClinicalTrials.gov Identifier: NCT00663546). Written informed consent was obtained from each participant. Written informed consent to participate in this study was provided by the participants' legal guardian/next of kin.

## Author Contributions

CC and PD conceptualized and supervised the study. CA, SSu, SSr, DP, LB, and RF coordinated the clinical study. CA, LB, and RF obtained the samples. BT performed the experiments. NM, NS, DN, and NT provided recombinant proteins. CC, BT, NA, and JK performed the analyses. All authors interpreted the data. BT, CC, and PD wrote the manuscript, with input from all authors.

### Conflict of Interest

The authors declare that the research was conducted in the absence of any commercial or financial relationships that could be construed as a potential conflict of interest.

## Supplementary Material

Table S1 Background data for all subjects from Brazil and Cambodia. Information on sex, age, hemoglobin levels, and parasitemia. Parasitemia data in Brazil, originally reported in semi-quantitative format, were converted to parasites per mm3 to be compared with data from Cambodia, reported as parasites/mm3. The conversions were: $\frac{1}{2}$+ = 50–300 parasites/mm3; + = 301–500 parasites per mm3; ++ = 501–10,000 parasites/mm3; +++ = 10,001–100,000 parasites.

Figure S1 Correlation between age and antibody titers against PvDBP-RII, PvCSP, and Pfs230D1M in Cambodia.

Figure S2 Confirmation of depletion (or reduction) of Pvs230D1M IgG from sera used to evaluate Pfs230D1M IgG levels.

Figure S3 Specificity of Pvs230D1M IgG depletion. PvDBP-RII IgG levels remain unaltered after depletion of Pvs230D1M IgG.
