# Supplementary figures and images for: Naturally Acquired Antibody Response to Malaria Transmission Blocking Vaccine Candidate Pvs230 Domain 1
Source: Front Immunol. 2019 Oct 4;10:2295. doi: 10.3389/fimmu.2019.02295 (PMC6788386; doi:10.3389/fimmu.2019.02295)

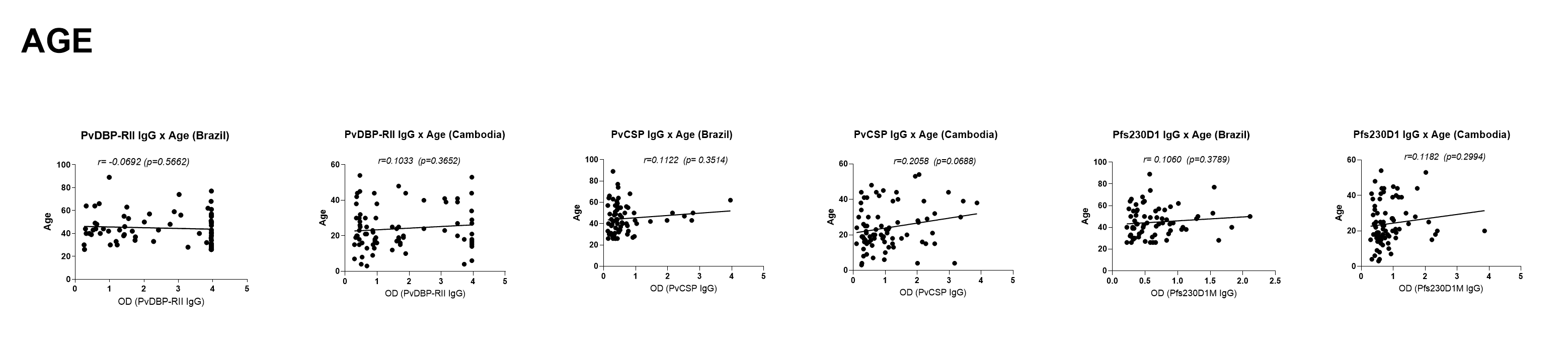

Supplement: Figure S1 — Correlation between age and antibody titers against PvDBP-RII, PvCSP, and Pfs230D1M in Cambodia. [file Image_1.tif]

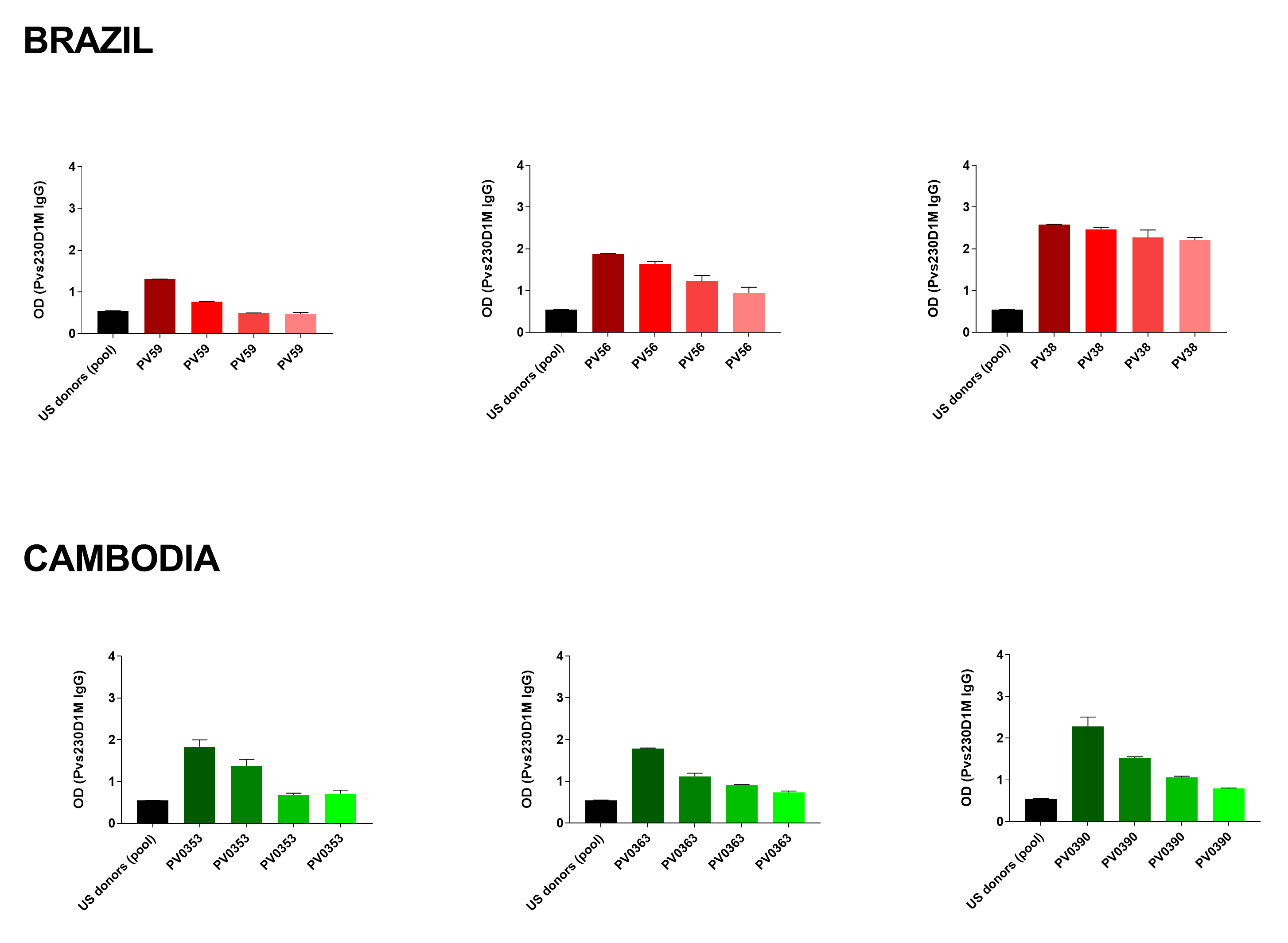

Supplement: Figure S2 — Confirmation of depletion (or reduction) of Pvs230D1M IgG from sera used to evaluate Pfs230D1M IgG levels. [file Image_2.tif]

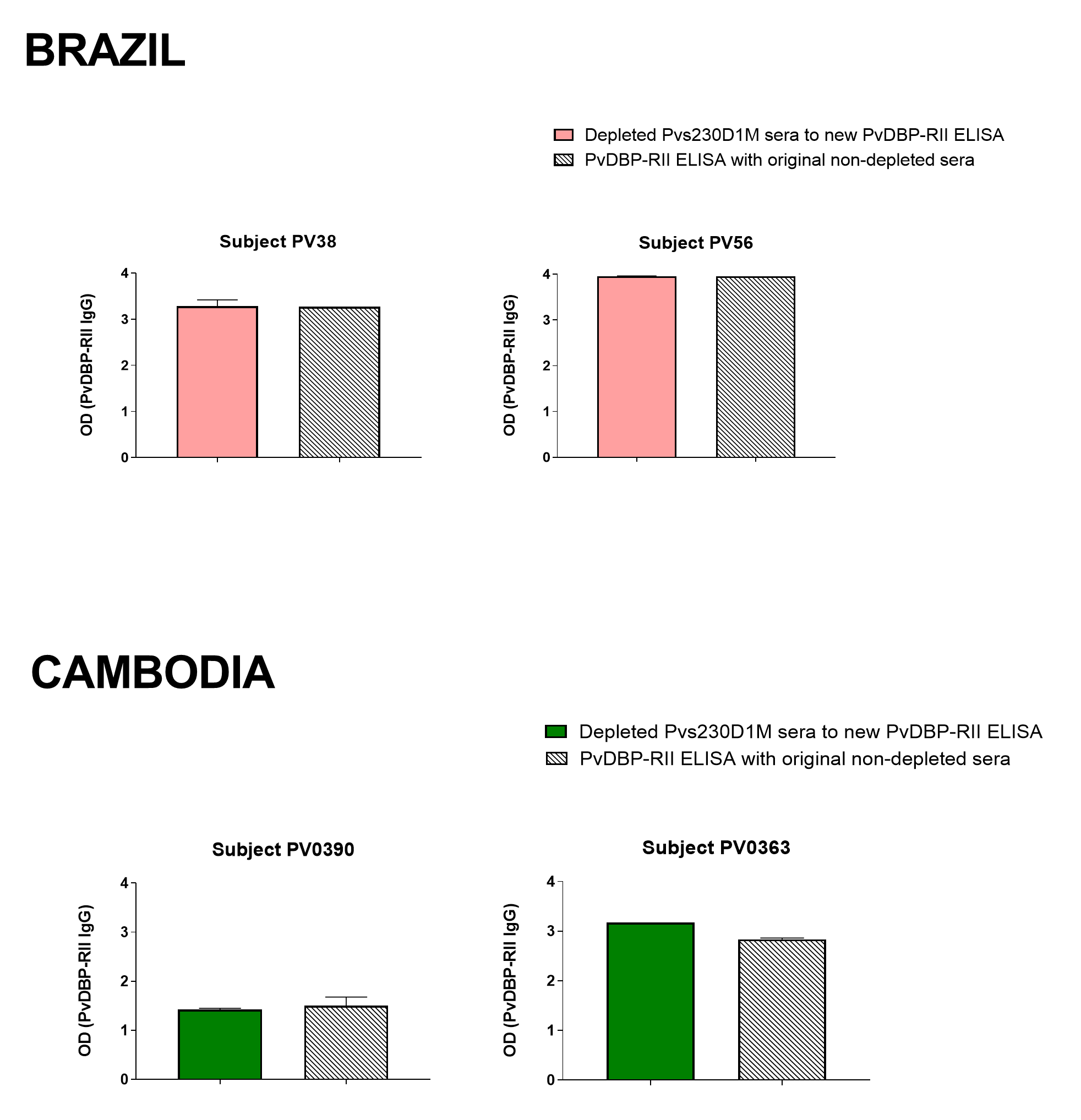

Supplement: Figure S3 — Specificity of Pvs230D1M IgG depletion. PvDBP-RII IgG levels remain unaltered after depletion of Pvs230D1M IgG. [file Image_3.tif]
